# Use of alternative and complementary therapies in labor and delivery care: a cross-sectional study of midwives’ training in Catalan hospitals accredited as centers for normal birth

**DOI:** 10.1186/1472-6882-13-318

**Published:** 2013-11-15

**Authors:** Ester Muñoz-Sellés, Antoni Vallès-Segalés, Josefina Goberna-Tricas

**Affiliations:** 1 Department of Public Health, Mental Health and Perinatal Nursing, University School of Nursing, University of Barcelona, Barcelona, Spain; 2 Department of Public Health, Faculty of Medicine, University of Barcelona, Barcelona, Spain

**Keywords:** Midwife, Complementary and alternative medicine (CAM), Training

## Abstract

**Background:**

The use of complementary and alternative medicine (CAM) and complementary and alternative therapies (CAT) during pregnancy is increasing. Scientific evidence for CAM and CAT in the field of obstetrics mainly covers pain relief in labor. Midwives are responsible for labor and delivery care: hence, their knowledge of CAM and CAT is important. The aims of this study are to describe the professional profile of midwives who provide care for natural childbirth in Catalan hospitals accredited as centers for normal birth, to assess midwives’ level of training in CAT and their use of these therapies, and to identify specific resources for CAT in labor wards.

**Methods:**

A descriptive, cross-sectional, quantitative method was used to assess the level of training and use of CAT by midwives working at 28 hospitals in Catalonia, Spain, accredited as public normal birth centers.

**Results:**

Just under a third of midwives (30.4%) trained in CAT after completion of basic training. They trained in an average of 5.97 therapies (SD 3.56). The number of CAT in which the midwives were trained correlated negatively with age (r = - 0.284; p < 0.001) and with their time working at the hospital in years (r = - 0.136; p = 0.036). Midwives trained in CAT considered that the following therapies were useful or very useful for pain relief during labor and delivery: relaxation techniques (64.3%), hydrotherapy (84.8%) and the application of compresses to the perineum (75.9%). The availability of resources for providing CAT during normal birth care varied widely from center to center.

**Conclusions:**

Age may influence attitudes towards training. It is important to increase the number of midwives trained in CAM for pain relief during childbirth, in order to promote the use of CAT and ensure efficiency and safety. CAT resources at accredited hospitals providing normal childbirth care should also be standardized.

## Background

Complementary and alternative medicine (CAM) comprises a very wide group of therapeutic practices that are not habitually used by conventional or allopathic health professionals [1-6].

The percentages of the population who report having used CAM at least once vary considerably, from 80% in Africa, 70% in Canada, 49% in France, 46% in Australia, 42% in the US, 40% in China, 31% in Belgium and 18% in Holland [7,8]. According to a study by the Spanish Ministry of Health’s Observatory of Natural Therapies, 95% of the Spanish population are aware of natural therapies and 23% have used one [8]. The US National Center for Complementary and Alternative Medicine (NCCAM) defines a complementary therapy as one that is used together with conventional medicine, whereas an alternative therapy is one used in the place of conventional medicine [9].

The term “integrative medicine” is used to refer to the combination of conventional and alternative medicine practices that meets the requirements of scientific evidence, safety and effectiveness [9-12]. Though regulations and funding vary from country to country, healthcare professionals are increasingly likely to prescribe alternative and complementary therapies (CAT) [13-15]. At the National Conference on Nursing and Medical Education, it was recommended that training for healthcare professionals should include CAM [16]. There is no consensus on current educational programs in different countries, but there is clearly a need to relate CAM training with evidence-based medicine [17-23]. In Spain, as CAM training for healthcare professionals is not regulated, their familiarity with the area varies widely [8]. CAM and CAT are offered as optional subjects at certain nursing schools, but not in a systematic way [24]; as a result, students do not gain a thorough grounding in the use of these therapies [11]. On qualifying, nurses who are interested in furthering their knowledge of CAM must seek postgraduate training available at certain universities or professional institutions [5-25]. In Spain, midwife training is a specialty of nursing [5]. In 2002, the International Confederation of Midwives published guidelines for basic midwifery knowledge, skills and behaviors that are essential to risk-free practice. Competency No. 4 includes comfort measures in labor, such as emotional support and non-pharmacological methods of pain relief [26]. In Spain, the current training program for midwives comprises two years of full time study, with a total of 3534 hours. Two thirds of the program is devoted to practical training in hospitals and outpatient centers. The theoretical training includes aspects such as the use of non-pharmacological techniques for pain relief during birth, including relaxation and breathing techniques, emotional support, techniques to promote mobility during labor, and the application of cold compresses to the perineum [5-26]. The amount of practical training in CAT given depends on the extent to which these treatments are used at the hospital where the student is training. Postgraduate training for midwives is usually shared with nurses and other health professionals. There are few specific training resources in CAM and CAT for trainee midwives.

Pettigrew (2002) stated that almost 70% of expectant mothers use some kind of CAM at the end of pregnancy, and that some of the practices tend to be self-prescribed [27]. Other studies suggest that the use of traditional medicine and CAT is not significantly associated with adverse obstetric events. [28] Scientific evidence for CAT in the field of obstetrics mainly covers pain relief in labor by relaxation and yoga [29] acupuncture, acupressure and hypnosis [30-32] warm water immersion during labor [33,34] and continuous support [35] as well as perineal pain relief through the application of cold compresses to the perineum [36]. There are contradictory recommendations on the use of the transcutaneous electrical nerve stimulation (TENS) technique – which is already applied in other clinical processes – for pain relief in the first stage of labor [32-37] and there is no consensus on the use of sterile water injections in the lumbar region [32-38]. More scientific evidence is required in these two areas. All these techniques are suitable for low-risk pregnancies and deliveries [25]. During the twentieth century, childbirth care in Spain became increasingly medicalized. However, pressure from women’s groups, associations and institutions led the health authorities to promote approaches to labor and delivery care that involve less intervention [34]. Various healthcare protocols and strategies have emerged to support normal, non-medicalized birth; improving the quality of professional care, taking a woman-centered approach, giving up harmful, unnecessary practices and maintaining current levels of safety [39-44]. The participation of the midwife in normal delivery care is central to all these strategies, and most of them include birth plan templates with various proposals for alternative pain relief methods (CAT). The idea is that expectant mothers should be able to choose from a range of tools that ease discomfort and pain during delivery [39,45,46] and so midwives must be familiar with CAT [47]. At the same time, health authorities have designed various strategic plans to improve the model of healthcare and to ensure that it reflects the philosophy of reducing the level of intervention. In 2007, the Spanish Ministry of Health and Consumer Affairs published a document entitled *Estrategia de atención al parto normal en el Sistema Nacional de Salud* (Strategy for Care in Normal Birth in the National Health System). The Spanish state is divided politically into Autonomous Communities, which have legislative and executive powers in the area of health. In Catalonia, which is one of these 17 autonomous regions, the regional Ministry of Health (the government body responsible for ensuring universal healthcare in Catalonia) has developed a policy of accrediting hospitals that are considered ready to provide care which respects the physiology of normal birth. The Ministry of Health provides subsidies for these hospitals to adapt their facilities, promote a training plan for professionals and acquire specific equipment. Recognized institutions are called “hospitals accredited for normal birth” and all of them belong to the public health system. In these hospitals, midwives are responsible for labor and delivery care. Hence, it is also in these hospitals that the importance of midwives’ knowledge and training in CAT or alternative methods of pain relief during birth is evident [8-48]. Therefore, the aim of this study was to establish the level of CAT training of midwives at hospitals accredited as centers for normal birth, and to determine the infrastructure and resources available in these hospitals to offer CAT to women during birth.

Our objectives were to describe the professional profile of midwives who work in delivery rooms in Catalan hospitals that are accredited as centers for normal birth; to determine the level of training and use of CAT by these midwives; and to identify specific resources in the labor wards of accredited Catalan hospitals.

## Methods

### Study design

The study was descriptive and cross-sectional, and a quantitative method was used.

### Study area

The study was conducted in Catalonia, where there are some 70 hospitals in total. We centered on the 28 public hospitals which, by 2011, had been accredited by the regional Ministry of Health as hospitals for normal birth.

### Subjects

The study population comprised the 455 midwives who work at these hospitals.

Midwives were included in the study if they had worked for at least six months at one of the hospitals. This was considered the minimum amount of time required to ensure that midwives had experience in applying the normal birth care protocol. Midwives who did not wish to take part in the study and midwives in training were excluded.

### Measuring instruments

A questionnaire drawn up by the research team was used to gather information from the midwives. It was based on two questionnaires compiled by Sara and Hastings-Tolsma (2009) [49,50]. The new questionnaire was adapted to the social, cultural and healthcare context and to the objectives of this study. The number of therapies under study was increased. Therapies were selected taking into account the results of studies in the literature on the CAT most frequently used during childbirth. In addition, questions on midwives’ knowledge and training in CAT were adapted to the Spanish educational system.

#### Description of the questionnaire

The questionnaire can be found in Additional file 1. In the first section, the midwives provided personal and professional information. The aim of the second section is to examine midwives’ training in CAT. In this section, midwives wrote down the number of hours of training they had received on each one of the proposed therapies, and stated when the training had taken place and the number of years’ experience they had in its use. The third section was designed to find out midwives’ perceptions of the degree of usefulness of CAT during labor and delivery, using a 5-point Likert scale. A specific table was drawn up for data on the material resources at the hospitals accredited as centers for normal birth. These data were supplemented by information from nursing supervisors on the labor wards (see Table 1). A pilot test of the questionnaire was carried out in November 2011 in order to revise contents and wording, and to estimate the completion time. The test was administered to ten midwives from one of the hospitals in the study, who completed the questionnaire twice in a fifteen-day period. Parts of the questionnaire that were difficult to understand were modified. The correlation between the two phases of questionnaire administration was measured using bivariate analysis. In the analysis of quantitative variables (Spearman’s rank correlation coefficient) and the analysis of qualitative variables (Pearson’s χ^2^ test), the correlations between the two questionnaires were higher than 88%.

**Table 1:** **Midwives training in complementary and alternative therapies**, **237 midwives**

	**N (%)**	**Training hours**	**Years of experience**
		**Average/SD**	**Average/SD**
**Deep-breathing exercises training**	180 (75.9)	30.76/53.72	10.11/9.23
**Relaxation techniques training**	176 (73.8)	33.29/53.88	10.59/8.99
**Continuous support training**	132 (55.7)	58.32/85.82	9.97/9.04
Postural therapy training	127 (53.6)	27.56/44.53	7.77/6.69
Application perineum compresses training	123 (51.9)	9.38/24.69	5.4/4.7
Massage training	108 (45.6)	35.59/65.86	7.71/6.71
Guided imagery training	101 (42.6)	21.02/26.81	8.79/8.07
Hydrotherapy training	88 (37.1)	23.24/36.34	5.18/5.27
Homeopathy training	72 (30.4)	16.25/16.94	2.83/1.51
Training in sterile water injections administration	66 (27.8)	5.18/5.5	3/2.23
Music therapy training	65 (27.4)	15.83/21.9	7.38/6.81
Reflex therapy training	60 (25.3)	43.74/61.91	5.47/5.67
Yoga training	41 (17.3)	88.86/158.18	5,57/6.23
Herbal medicine training	41 (17.3)	44.68/76.68	4.91/5.62
Therapeutic touch training	40 (16.9)	25.38/22.37	6.31/7.13
Transcutaneous electrical nerve stimulation (TENS)	38 (16)	5.81/7.21	3.9/2.35
Aromatherapy training	32 (13.5)	15.4/15.23	4.78/4.48
Flower essences training flower essences	29 (12.2)	31.44/24.65	4.49/3.9
Acupressure training	23 (9.7)	57.8/85.61	3.9/2.4
Acupuncture training	19 (8)	110/134.4	5.12/8.14
Hypnosis training	18 (7.6)	18.77/22.96	5.3/6.73
*Reiki training*	13 (5.5)	41/74.84	8,25/6.18
*Ear puncture training*	8 (3.4)	91.43/112.46	8/11.4
*Orthomolecular medicine training*	4 (1.7)	33.5/25.6	2.75/2.36

Midwives are trained in different therapies. The best training therapy is breathing technics 75.9% and the worse is orthomolecular medicine 1.7%. In bold the 3 therapies more training *in italics the 3 worse*.

### Statistical analysis

We conducted a descriptive analysis of the midwives’ sociodemographic and professional variables, variables related to the hospitals in the study, and variables referring to CAT training. Descriptive statistics were summarized using the frequencies (percentages) for categorical variables. Means, standard deviations, medians, minimums and maximums were reported for all continuous variables. To compare variables, we used X^2^ for categorical variables, the student’s t-test for independent groups for categorical variables with two categories that met the applicability criteria, assuming equal variance, and the Pearson’s correlation for two quantitative variables. A value of p < 0.05 was considered to be significant. The software used for the analysis was SPSS Statistics v20.

### Ethical aspects

This study was approved by the University of Barcelona Bioethics Committee and the Ethics and Clinical Research Committee of the first two hospitals where data were compiled. The other hospitals authorized the study after examining the project and after being notified of the authorization granted by the Bioethics Committees of the University and the two first hospitals. An information session was held in each of the participating hospitals to explain the study aims and to request collaboration. At the end of the talk, a copy of the questionnaire was given to each midwife, along with an informed consent form. The nursing supervisor at each of the hospitals was in charge of keeping the questionnaires until the research team collected them two weeks later.

## Results

Of the 465 questionnaires administered at the start of the study, 237 were returned fully completed. The overall response rate was 54.06%. The response rate for different hospitals varied between 100% and 13.33%.

All of the nursing supervisors were women, with an average age of 46.82 years (SD 7.9). Of the midwives, 3.4% were male and 96.6% were female and the average age was 41.21 years (SD 10.28). A total of 75.1% (n = 178) worked full-time and 18.4% (n = 43) part-time. A total of 76.4% (n = 181) had trained in Catalonia, 3.8% (n = 9) in the rest of Spain, 9.3% (n = 22) in South America and 9.3% (n = 22) in the rest of Europe. On average, midwives had worked for 14.64 years in the specialty (SD 12.3); 9.4 years in delivery care at their hospital (SD 8.9); and 6.2 years in care for normal childbirth (SD 7.38). Of the midwives surveyed, 89.87% (n = 213) stated that they had trained in some form of CAT. On average, they had trained in 7.58 of the therapies under study (SD 4.21; minimum 1, maximum 23). If we exclude therapies that already form part of basic midwifery training, the percentage of midwives who trained in CAT after completion of basic training was 30.4% (n = 72), in 5.97 therapies on average (SD 3.56; minimum 1, maximum 18). Midwives were mainly trained in breathing techniques 75.9% (n = 180), relaxation techniques 73.8% (n = 176), continuous support 55.7% (n = 132), postural therapy 53.6% (n = 127) and the application of compresses to the perineum 51.9% (n = 123). Very few midwives were trained in ear acupuncture 3.4% (n = 8) and orthomolecular medicine 1.7% (n = 4) (see Table 2). Midwives trained in CAT considered that the following therapies were quite or very useful for pain relief during labor and delivery: relaxation techniques, hydrotherapy and the application of compresses to the perineum. However, they considered that the following CAT were hardly or not at all useful: acupuncture needles, sterile water injections in the lumbar region, hypnosis and TENS (see Table 2). A total of 39.3% of the midwives worked in a level I hospital (low technical complexity), 28.6% in a level II hospital (average technical complexity) and 32.1% in a level III hospital (high technical complexity). In Catalonia, there were 79,820 deliveries in 2011, of which 44,481 took place at the hospitals under study. The average number of births per hospital was 1594.04 (SD 934.9). A total of 6,075 women requested care for natural childbirth, but only 4,752 of them received such care. The percentages of natural births ranged from 71.51 to 5.66% at the Catalan hospitals under study. The hospital resources for providing care during normal births using CAT varied widely (see Table 3). We studied midwives’ perceptions of the usefulness of CAT for pain relief during labor and birth. No statistically significant differences were found for gender, specialty training school, length of time that the hospital had been accredited as a normal birth center, type of work contract, length of time since training in the specialty, level of care provided by the hospital and years of experience in providing care for normal delivery *versus* the number of CAT in which the midwives had trained, as well as the hours and total years of training in the CAT under study. The total number of CAT in which the midwives were trained correlated negatively with age (r = - 0.284; p < 0.001) and with the years in which midwives had been working at the hospital (r = - 0.136; p = 0.036).

**Table 2:** Perceived usefulness of Scientific evidence recommended therapies

**For pain relief in labour**		**Nothing or any**	**Regular**	**Rather or much**	**Total:**
Acupuncture needles applications	N (%)	10(55.5)	5 (27.7)	8 (44.4)	18 (100)
Perineum compresses		3 (3.6)	17 (20.5)	63 (75.9)	83 (100)
Hydrotherapy		7 (8.8)	5 (6.3)	67 (84.8)	79 (100)
Hypnosis		2 (20)	3 (30)	5 (50)	10 (100)
Water injections		8 (34.7)	5 (21.7)	10 (43.4)	23 (100)
Massage		4 (4.8)	18 (21.4)	62 (73.8)	84 (100)
Relaxation techniques		7 (5)	43 (30.7)	90 (64.2)	140 (100)
Deep-breathing exercises training		6 (3.8)	44 (28)	107 (68.1)	157 (100)
Postural therapy		2 (1.8)	18 (15.9)	92 (82.1)	112 (100)
Transcutaneous electrical nerve stimulation		8 (38)	4 (19)	9 (42.8)	21 (100)

**Table 3:** Hospital resources of the 28 hospitals studied in reference to natural birth

**Classification**	**Therapies**		**Resource owned hospitals. N (%)**	**Number of hospitals that allow women bring home her remedy. N (%)**
Biological techniques		Phytotherapy	4 (14.3)	2 (7.14)
		Orthomolecular medicine	0	
Mind-body medicine	Hydrotherapy	Bath	12 (42.9)	
		Hot water bottles	21 (75)	
		Shower	27 (96.4)	
	Music therapy	Radio-cd	22 (78.6)	2 (7.14)
		Perineum compresses	24 (85.7)	
	Postural therapy	Birth bed	27 (96.4)	
		Balls	28 (100)	
Whole medical systems		Homeopathy	3 (10.7)	5 (17.85)
		Acupuncture needles	0	
Manipulative and body-based practices		Water injections	12 (42.9)	
	Massage aromatherapy reflex therapy	Massage oils	8 (28.6)	3 (10.7)
		Essentials oils	4 (14.3)	3 (10.7)
	Transcutaneous electrical nerve stimulation	TENS	9 (32.1)	
Energetic therapies		Bach remedies	1 (3.6)	4 (14.28)

## Discussion

The response rate in this study was 54.06%, which is similar to that found in other studies [51]. One explanation for the high non-response rate is that midwives with no CAT training did not complete the questionnaire. This, together with the fact that the questionnaire was only administered in hospitals accredited as centers for normal birth, could explain why midwives in this study had a high percentage (89.87%) of CAT training. This result corroborates the findings of Bjerså (2012), who observed that 80% of nurses had CAT training [52]. The percentage of training is lower in other studies. Forcades (2004) reported that 54% of cases medical students consider that they have sufficient knowledge of alternative medicines [53]. Shorofi (2010) stated that 40% of nurses were not trained in CAT [54]. In Catalonia, the Nurses’ Association of Barcelona considers that 25.5% of nurses use alternative therapies [11] and Fernandez (2010) found 58.8% of nurses who care for cancer patients have CAT training [55]. The basic midwifery training program in Spain includes relaxation and breathing techniques, position changes and continuous support for women during labor and birth. These techniques are recommended for all women, whether they want a more natural or more medicalized birth. When we excluded these techniques from the total number of alternative therapies in which midwives in this study were trained, we found that only 30.4% (72) had training in other *more specific* CAT. These data are similar to those found in a study carried out in Germany by Wiebelitz (2009) [56]. Midwives’ opinions about the use of CAT differed: Some considered that more solid evidence was required to recommend them, whilst others believed that they were useful and safe during pregnancy [27-57]. Midwives’ perceptions of the utility of CAT may be due to the fact that they were unaware of the institutional recommendations on pain relief techniques and had little experience in their application. The results show a negative correlation with age and midwives’ years of work at the hospital: midwives who were older and had more years of experience tended to have less CAT training [58]. Indeed, age may influence attitudes towards training [59].

## Conclusions

It is important to reach a consensus on the definitions of normal birth, so that we can compare data from different hospitals. The results of this study indicate that some midwives are trained in CAT but cannot apply the therapies, due to a lack of specific equipment or insufficient resources in their hospitals. Consequently, more resources should be made available for the use of CAT in normal birth care, in terms of training, infrastructure and equipment. CAT should be included in the care protocols for labor wards, so that professionals can use them in their daily work. In 1995, the WHO stressed the importance of qualifications for CAT professionals and providers, and the need to incorporate CAT in the training of healthcare professionals, so that they would become qualified to offer these therapies. Current training depends on individual initiative. Therefore, it may be affected by a lack of knowledge of CAT, a lack of initial training, little interest in the subject and insufficient resources. It is important to stress to midwives the importance of CAT training, so that they can provide suitable care for women who want to use these therapies. Therefore, it is essential to increase the courses for midwives so that they can broaden their knowledge of CAT for birth and provide effective care for women. Health authorities must ensure safe interventions and good practices, incorporate CAT into courses for health care professionals and into the health system, and contribute to standardizing the effective and safe use of CAT.

## Abbreviations

CAM: Complementary and alternative medicine; CAT: Complementary and alternative therapies. In this study, the concept of CAT includes all non-pharmacological techniques for pain relief during birth; TENS: Transcutaneous electrical nerve stimulation.

## Competing interests

None of the authors had competing interests. This study was funded by a grant (PREUI 12/06) from the Research Committee of the UB University School of Nursing.

## Authors’ contributions

EMS contributed substantially to the conception and design of the study, the collection of data, the compilation of results and the final report. This study is part of EMS’s doctoral thesis. AVS helped to validate the questionnaire and assisted in the statistical analysis of data. JGT supervised the design of the study, contacted some of the hospitals to obtain authorization to gather data, sought funding for the study and made considerable intellectual contributions to the revision of the manuscript. All the authors read and approved the final version of the manuscript.

## Pre-publication history

The pre-publication history for this paper can be accessed here:

http://www.biomedcentral.com/1472-6882/13/318/prepub

## Supplementary Material

Additional file 1 Questionnaire for midwives. Click here for file

## References

[B1] Allison N The illustrated encyclopedia of body-mind disciplines 1999 New York: Rosen Pub Group

[B2] Wieland L Manheimer E Berman B Development and classification of an operational definition of complementary and alternative medicine for the Cochrane collaboration Altern Ther Health Med 2011 17 2 50 59 21717826 PMC3196853

[B3] Adams J Sibbritt D Broom A Loxton D Pirotta M Humphreys J A comparison of complementary and alternative medicine users and use across geographical areas: A national survey of 1,427 women BMC Complement Altern Med 2011 7 11 85 21981986 10.1186/1472-6882-11-85 PMC3198987

[B4] Gentz B Alternative therapies for the management of pain in labor and delivery Clin Obstet Gynecol 2001 44 4 704 732 10.1097/00003081-200112000-00010 11600853

[B5] Orden SAS/1349/2009 de 6 de mayo, por la que se aprueba y publica el programa formativo de la especialidad de Enfermería Obstétrico-Ginecológica (Matrona) http://www.boe.es/buscar/doc.php?id=BOE-A-2009-8881

[B6] Adams J Lui C Sibbritt D Broom A Wardle J Homer C Attitudes and referral practices of maternity care professionals with regard to complementary and alternative medicine: an integrative review J Adv Nurs 2011 67 3 472 483 10.1111/j.1365-2648.2010.05510.x 21214615

[B7] Adams J An exploratory study of complementary and alternative medicine in hospital midwifery: Models of care and professional struggle Complement Ther Clin Pract 2006 12 40 47 10.1016/j.ctcp.2005.09.003 16401529

[B8] Ministerio de sanidad, política social e igualdad Terápias naturales http://www.msssi.gob.es/novedades/docs/analisisSituacionTNatu.pdf

[B9] National Center for Complementary and Alternative Medicine (NCCAM) What is CAM? http://nccam.nih.gov/health/whatiscam

[B10] Duarte M Medicina occidental y otras alternativas: ¿es posible su complementariedad? reflexiones conceptuales Cad Saude Publica 2003 19 2 635 643 10.1590/S0102-311X2003000200030 12764479

[B11] López-Ruíz J Arqué-Blanco M Bases per a l’acreditació de la competència dels professionals infermers en teràpies naturals i complementàries 2009 Barcelona: Col · legi Oficial d’infermeria de Barcelona

[B12] Sibbritt D Adams J Developing and promoting public health methods for integrative medicine: Examples from the field in Australia Zhong Xi Yi Jie He Xue Bao 2011 9 3 233 36 10.3736/jcim20110301 21419073

[B13] Lázaro M Terapias alternativas y complementarias: evidencia clínica SEDENE 2008 27 12 26

[B14] O’Regan P Wills T The growth of complementary therapies: and their benefits in the perioperative setting J Perioper Pract 2009 19 11 386 389 10.1177/175045890901901102 20041625

[B15] Hirschkorna K Bourgeaulta I Actions speak louder than words: mainstream health providers’ definitions and behaviour regarding complementary and alternative medicine Complement Ther Clin Pract 2007 13 29 37 10.1016/j.ctcp.2006.05.001 17210509

[B16] Gaylord S Mann D Rationales for CAM education in health professions training programs Acad Med 2007 82 927 933 10.1097/ACM.0b013e31814a5b43 17895650

[B17] Connelly E Elmer P Morris C Zwickey H The vanguard faculty program: research training for complementary and alternative medicine faculty J Altern Complement Med 2010 16 10 1117 1123 10.1089/acm.2009.0574 20874443 PMC3110828

[B18] Ezzo J Wright K Hadhazy V Bahr-Robertson M Mc Beckner W Covington M Use of the cochrane electronic library in complementary and alternative medicine courses in medical schools: is the giant lost in cyberspace? J Altern Complement Med 2002 8 5 681 686 10.1089/107555302320825200 12470450

[B19] Gaster B Unterborn J Scott R Schneeweiss R What should students learn about complementary and alternative medicine? Acad Med 2007 82 934 938 10.1097/ACM.0b013e318149eb56 17895651

[B20] Marcus D McCullough L An evaluation of the evidence in “Evidence-based” integrative medicine programs Acad Med 2009 84 1229 1234 10.1097/ACM.0b013e3181b185f4 19707062

[B21] Armi H Haramati A Basic science to develop an innovative program in complementary and alternative medicine J Int Assoc Med Sci Educ 2010 20 2 48 55 21243105 PMC3019605

[B22] Lee M Ben R Wimsatt L Cornman J Hedgecock J Gerik S Integrating complementary and alternative medicine instruction into health professions education: Organizational and instructional strategies Acad Med 2007 82 939 945 10.1097/ACM.0b013e318149ebf8 17895652

[B23] Pearson N Chesney M The CAM education program of the national complementary and alternative medicine: An overview Acad Med 2007 82 921 926 10.1097/ACM.0b013e31814a5014 17895649

[B24] Rodríguez M Rojas M Abreu A Rodríguez J Enfermería y el presente de las terapias complementarias Rev ROL Enferm 2002 25 4 248 252 14502940

[B25] Muñoz-Sellés E Goberna-Tricas J Oferta formativa en terapias alternativas y complementarias para la asistencia al parto Matronas Prof 2012 13 2 50 54

[B26] International Confederation of Midwives Essential competencies for basic midwifery practicehttp://www.internationalmidwives.org/what-we-do/education-coredocuments/essential-competencies-basic-midwifery-practice/10.1016/j.midw.2011.03.005 21601321

[B27] Ernst E Watson L Midwives’ use of complementary/alternative treatments Midwifery 2012 28 6 772 777 10.1016/j.midw.2011.08.013 22015222

[B28] Mureyi D Monera T Maponga C Prevalence and patterns of prenatal use of traditional medicine among women at selected harare clinics: A cross-sectional study BMC Complement Altern Med 2012 12 164 10.1186/1472-6882-12-164 23016608 PMC3533916

[B29] Smith C Levett K Collins C Crowther C Relaxation techniques for pain management in labor Cochrane Database Syst Rev 2011 CD009514 12 10.1002/14651858.CD009514 10.1002/14651858.CD009514 22161453

[B30] Smith C Collins C Crowther C Levett K Acupuntura o acupresión para el tratamiento del dolor durante el trabajo de parto Cochrane Database Syst Rev 2011 CD009232 7 10.1002/14651858.CD009232

[B31] Smith C Collins C Cyna A Crowther C Tratamientos complementarios y alternativos para el manejo del dolor durante el trabajo de parto (revisión cochrane traducida) La Biblioteca Cochrane Plus 2008 4 Oxford Update Software Ltd. http://www.bibliotecacochrane.com/BCPGetDocument.asp?SessionID=%207819852&DocumentID=CD003521

[B32] Slade S Labor: Non Pharmacological Pain Relief. In Joanna Brigs Institute. Evidence Summarie. COnNECT+ http://connect.jbiconnectplus.org/Search.aspx

[B33] Cluet E Nikodem V McCandilish R Burns E Inmersión en agua para el embarazo, trabajo de parto y parto; (revisión cochrane traducida) 2008 2 Oxford: La biblioteca Cochrane plus Update Software Ltd. [http://www.bibliotecacochrane.com/BCPGetDocument.asp?SessionID=%207819852&DocumentID=CD000111 (translated from The Cochrane Library, 2008 Issue 2. Chichester, UK: John Wiley & Sons, Ltd]

[B34] Goberna-Tricas J Dones i procreació: Ètica de les pràctiques sanitàries i la relació assistencial en l’embaràs i naixement. (PhD thesis) 2009 Barcelona: Universitat de Barcelona http://hdl.handle.net/10803/1762

[B35] Hodnett E Gates S Hofmeyr G Sakala C Apoyo continuo para las mujeres durante el parto (revisión cochrane traducida) La Biblioteca Cochrane Plus 2008 4 Oxford Update Software Ltd [http://www.bibliotecacochrane.com/BCPGetDocument.asp?SessionID=%207819852&DocumentID=CD003766. (Translated from The Cochrane Library, 2008 Issue 3. Chichester, UK: John Wiley & Sons, Ltd]

[B36] East C Begg L Henshall N Marchant P Wallace K Frío local para el alivio del dolor producido por el trauma perineal prolongado durante el parto (revisión cochrane traducida) La Biblioteca Cochrane Plus 2008 4 Oxford Update Software http://www.bibliotecacochrane.com/BCPGetDocument.asp?SessionID=%207819852&DocumentID=CD006304

[B37] Dowswell T Bedwell C Lavender T Neilson J Estimulación nerviosa eléctrica transcutánea (ENET) para el alivio del dolor durante el trabajo de parto (revision cochrane traducida) La Biblioteca Cochrane Plus 2009 3 Oxford Update Software Ltd http://www.bibliotecacochrane.com/BCPGetDocument.asp?SessionID=%207819852&DocumentID=CD007214

[B38] Derry S Straube S Moore R Hancock H Collins S Intracutaneous or subcutaneous sterile water injection compared with blinded controls for pain management in labor Cochrane Database Syst Rev 2012 18 1 CD009107 doi: 10 1002/14651858 CD009107 pub2 22258999 10.1002/14651858.CD009107.pub2 PMC11663508

[B39] Departament de Salut Protocol per a l’assistència natural al part normal 2007 Barcelona: Direcció General de Salut Pública. Generalitat de Catalunya

[B40] Observatorio de Salud de la Mujer y del Sistema Nacional de Salud. Dirección General. Agencia de Calidad del Sistema Nacional de Salud Estrategia de atención al parto normal en el Sistema Nacional de Salud 2008 Madrid: Ministerio de sanidad y consumo

[B41] Ministerio de sanidad, política social e igualdad Estrategia Nacional de Salud Sexual y Reproductiva 2011 Madrid: Ministerio de Sanidad, política social e igualdad

[B42] FAME Iniciativa parto normal. Documento de consenso 2007 Barcelona: Federación de Asociaciones de Matronas de España

[B43] Ministerio de Sanidad, política social Guía de Práctica Clínica sobre la Atención al Parto Normal 2010 Vitoria-Gasteiz: Eusko Jaurlaritzaren Argitalpen Zerbitzu Nagusia. Servicio Central de Publicaciones del Gobierno Vasco

[B44] Agencia de Calidad del Sistema Nacional de Salud. Observatorio de salud de las mujeres Plan de Parto y Nacimiento. Estrategia de atención al parto normal 2010 Madrid: Ministerio de Sanidad, Política Social e Igualdad

[B45] Bailey J Crane P Nugent C Childbirth education and birth plans Obstet Gynecol Clin North Am 2008 35 3 497 509 10.1016/j.ogc.2008.04.005 18760232

[B46] Lothian J Birth plans: The good, the bad, and the future JOGNN 2006 35 2 295 303 16620258 10.1111/j.1552-6909.2006.00042.x

[B47] Dayhew M Wilkinson J Simpson M Complementary and alternative medicine and the search for knowledge by conventional health care practitioners Contemp Nurse 2009 33 1 41 49 10.5172/conu.33.1.41 19715494

[B48] Departament de Salut El sistema de salut i la xarxa sanitària pública de catalunya. línies estratègiques i evolució 2004-2009 2009 Barcelona: Generalitat de Catalunya

[B49] Hastings-Tolsma M Terada M Complementary medicine use by midwives in the U.S Complement Ther Clin Pract 2009 15 212 19 10.1016/j.ctcp.2009.06.016 19880084

[B50] Quandt SA Verhoef MJ Arcury TA Lewith GT Steinsbekk A Kristoffersen AE Development of an international questionnaire to measure use of complementary and alternative medicine (I-CAM-Q) J Altern Complement Med 2009 15 4 331 9 10.1089/acm.2008.0521 19388855 PMC3189003

[B51] Fincham J Response rates and responsiveness for surveys, standards, and the journal Am J Pharm Educ 2008 15 2 43 72 18483608 10.5688/aj720243 PMC2384218

[B52] Bjersa K Victorin E Olsén M Knowledge about complementary, alternative adn integrative medicine (CAM) among registered health care providers in swedish surgical care: A national survey among university hospitals BMC Complement Altern Med 2012 12 42 10.1186/1472-6882-12-42 22498305 PMC3373365

[B53] Forcades T Estudi observacional de l’impacte de les medicines alternatives en els estudiants de medicina de catalunya. (PhD thesis) 2004 Barcelona: Universitat de Barcelona

[B54] Shorofi S Arbo P Nurses’ knowledge, attitudes, and professional use of complementary and alternative medicine (CAM): A survey at five metropolitan hospitals in adelaide Complement Ther Clin Pract 2010 16 229 234 10.1016/j.ctcp.2010.05.008 20920809

[B55] Fernandez A Salvador T Formación y aplicación de las terapias complementarias en los cuidados de enfermería al paciente oncológico Nursing 2010 28 7 52 58

[B56] Wiebelitz K Goecke T Brach J Beer A Use of complementary and alternative medicine in obstetrics Br J Midwifery 2009 17 3 169 175

[B57] Stone J Using complementary therapies within nursing: Some ethical and legal considerations Complement Therap Nurs Midwifery 1999 5 46 50 10.1016/S1353-6117(99)80054-7 10474347

[B58] Fournier C Formar a los empleados de mayor edad: Un objetivo a reformular Calificaciones&empleo 2011 78 1 4

[B59] Samuels N Zisk-Rony R Singerm SR Use of and attitudes toward complementary and alternative medicine among nurse-midwives in Israel Am J Obstet Gynecol 2010 203 341 347 20541732 10.1016/j.ajog.2010.05.001

