# Supplementary material for: Use of alternative and complementary therapies in labor and delivery care: a cross-sectional study of midwives’ training in Catalan hospitals accredited as centers for normal birth
Source: BMC Complement Altern Med. 2013 Nov 15;13:318. doi: 10.1186/1472-6882-13-318 (PMC4225673; doi:10.1186/1472-6882-13-318)
Supplement: Additional file 1 — Questionnaire for midwives. [file 1472-6882-13-318-S1.pdf]

Additional files

Additional file 1 – QUESTIONNAIRE FOR MIDWIVES:

Personal and professional Data:

Gender: □ male □ female

Age: _______ years

Place where you studied midwifery specialty: ________________________

At what year did you end your training? ___________

How long have you worked in this hospital? _________ years

How long have you work in natural birth? _________ years

Training in complementary and alternative therapies:

	Duration	During nurse training		Before nurse training		Continuous training		Years of experience
Training in:	Nº hours	Yes	No	Yes	No	Yes	No	Nº years
**NATURAL PRODUCTS:**								
Phitotherapy								
Bach remedies								
Orthomolecular medicine								
**MIND-BODY MEDICINE:**								
Continued support								
Hydrotherapy								
Hypnosis								
Music therapy								
Relaxation techniques								
Deep-breathing exercises								
Guided imagery								
Postural therapy								
Yoga								
**WHOLE MEDICAL SYSTEMS:**								
Acupuncture								
Aromatherapy								
Ear puncture								
Homeopathy								
**MANIPULATIVE AND BODY-BASED PRACTICES:**								
Acupressure								
Perineum compresses								
Transcutaneous electrical nerve stimulation (TENS)								
Sterile water injection								
Massage								
Reflex therapy								
Shiatsu								
**ENERGETIC THERAPYES:**								
Reiki								
Therapeutic touch								
Others:								

Use of therapies during labour:

Mark with X your opinion for every therapy indication, next, tell us your opinion on their usefulness from 1 (useless) to 5 (very useful).

Use of therapies	Number of women		Indications	Use utility 1 nothing	2 any	3 regular	4 rather	5 much
**NATURALS PRODUCTS:**								
Phitotherapy		□	stimulation uterine contractions					
		□	promote cervical dilation					
		□	others:					
Bach remedies		□	reduce fears					
		□	reduce anxiety					
		□	improve foetus wellbeing					
		□	reduce discouragement					
		□	others:					
Orthomolecular medicine		□	stimulation uterine contractions					
		□	others:					
**MIND-BODY MEDICINE:**								
Continued support		□	reduce anxiety					
		□	others:					
Hydrotherapy		□	pain relief					
		□	promote cervical dilation					
		□	others:					
Hypnosis		□	pain relief					
		□	others:					
Music therapy		□	promote relaxation					
		□	improve concentration					
		□	others:					
Relaxation techniques		□	pain relief					
		□	others:					
Deep-breathing exercises		□	pain relief					
		□	others:					
Guided imagery		□	pain relief					
		□	others:					
Postural therapy		□	pain relief					
		□	promote cervical dilation					
		□	change foetus presentation					
Yoga		□	promote relaxation					
		□	improve concentration					
		□	others:					
**WHOLE MEDICAL SYSTEMS**								
Acupuncture/ Ear puncture		□	pain relief					
		□	stimulation uterine contractions					
		□	promote cervical dilation					
		□	others:					
Aromatherapy		□	stimulation uterine contractions					
		□	reduce anxiety					
		□	others:					
Homeopathy		□	stimulation uterine contractions					
		□	others:					

**MANIPULATIVE AND BODY-BASED PRACTICES**								
Perineum compress		☐	pain relief					
		☐	reduce perineum trauma					
Transcutaneous nerve stimulation		☐	pain relief					
Sterile water injections		☐	pain relief					
Massage		☐	pain relief					
		☐	reduce anxiety					
		☐	others:					
Reflex therapy		☐	stimulation uterine contractions					
		☐	others:					
**ENERGETIC THERAPIES**								
Reiki		☐	reduce anxiety					
		☐	others:					
Therapeutic touch		☐	reduce anxiety					
		☐	others:					
other:		☐	…………………………………...					
		☐	…………………………………...					

Additional file 2 –QUESTIONAIRE FOR NURSING SUPERVISORS ON THE LABOUR WARDS.

Personal and professional Data

Gender: □ Male □ Female

Age: _______ years

Professional Category: ______________

How long have you been working in this labour area? _______

Currently do you assist labours? __________

Number of parts of the hospital during 2011: _________

Number of parts for normal birth in 2011: _________

Hospital resources

**Hospital resources:**	Yes	No
Acupuncture needles		
Bath		
Hot water bottles		
Perineum compresses		
Shower		
Transcutaneous nerve stimulation (TENS)		
Phytotherapy		
Bach Remedies		
Homeopathy		
Sterile water injections		
Birthing bed		
Orthomolecular medicine		
Massage oils		
Essential Oils		
Balls		
Radio or CD for music therapy		
Other (specify):		
